# pAc/Emm55 increases anti-melanoma immunity via TLR2 signaling in dendritic cells and improves checkpoint blockade efficacy

**DOI:** 10.3389/fimmu.2026.1863894

**Published:** 2026-08-19

**Authors:** Thi Hong Nga Le, Quang Tam Nguyen, Youngchul Kim, Vishanna Balkaran, Trisha Bhathivi, Shari Pilon-Thomas, Joseph Markowitz

**Affiliations:** 1 Department of Cutaneous Oncology, H. Lee Moffitt Cancer Center and Research Institute, Tampa, FL, United States; 2 Biostatistics and Bioinformatics, H. Lee Moffitt Cancer Center and Research Institute, Tampa, FL, United States; 3 The Immuno-Oncology Program, H. Lee Moffitt Cancer Center and Research Institute, Tampa, FL, United States; 4 Immunology, H. Lee Moffitt Cancer Center and Research Institute, Tampa, FL, United States; 5 Center for Immunization and Infection Research in Cancer (CIIRC), H. Lee Moffitt Cancer Center and Research Institute, Tampa, FL, United States; 6 The Department of Oncologic Sciences, University of South Florida Morsani School of Medicine, Tampa, FL, United States

**Keywords:** antibody response, dendritic cells, IFx-Hu2.0, immune checkpoint blockade, melanoma, MyD88, pAc/Emm55, TLR2

## Abstract

**Background:**

pAc/Emm55 (IFx-Hu2.0) is a plasmid DNA therapy encoding a *Streptococcus pyogenes*-derived bacterial gene. It shows anti-tumor activity in preclinical models, but the mechanisms of anti-melanoma immunity and additive effects with immune checkpoint blockade remain unclear.

**Methods:**

B16 melanoma tumor-bearing mice were treated intratumorally with pAc/Emm55 alone and/or intraperitoneally with PD-1, CTLA-4, or LAG-3 blockade and monitored for tumor growth. Antibodies against Emm55 and melanoma cells (B16 or YUMM) were quantified by flow cytometry. Bone marrow-derived dendritic cells (BMDCs) from WT, MyD88 KO, TLR2 KO, or TLR7 KO mice were assessed for antigen uptake, OVA cross-presentation, and OT-I priming after pulsing with cell lysates from Emm55- or empty-transfected melanoma cells. Tumor immune infiltration by CD8 and CD11c cells were evaluated by immunohistochemistry.

**Results:**

IFx-Hu2.0 significantly reduced tumor growth, correlating with increased intratumoral CD8^+^ T cell infiltration and enhanced systemic antibody responses against melanoma cells. TLR deficiency did not alter OVA uptake or basal BMDC cross-presentation. However, Emm55-enhanced OT-I priming and IFN-γ production required TLR2 and MyD88, but not TLR7, supporting a TLR2–MyD88–dependent mechanism. Emm55 monotherapy and anti–PD-1 each reduced tumor burden. The combination further improved inhibition. Adding anti-CTLA-4 or anti-LAG-3 to anti–PD-1 did not provide additional meaningful benefit.

**Conclusions:**

Emm55 facilitates the TLR2–MyD88 pathway in DCs and subsequent IFN-γ production by OT-I CD8 T cells. Combining intratumoral IFx-Hu2.0 with PD-1 blockade improves tumor control, supporting clinical evaluation of this strategy as also suggested by the first-in-human clinical trial of IFx-Hu2.0.

## Introduction

Intratumoral immunotherapy aims to harness local immune activation within the tumor microenvironment to generate both localized and systemic antitumor responses while minimizing off-target effects and decreasing systemic toxicity. Melanoma is well-suited for this approach because many lesions are accessible for direct injections, and local immune priming can be paired with systemic immune checkpoint blockade (1, 2). In addition, intratumoral therapies such as talimogene laherparepvec have received FDA approval (3). However, frequent primary resistance and acquired progression on current front-line therapies highlight the need for new immune-priming strategies (4).

Immune checkpoint blockade, particularly PD-1 blockade, has transformed melanoma treatment. Durable clinical benefit is achieved in only a subset of patients (5, 6). Combinations such as anti-PD-1 plus anti-CTLA-4 or anti-LAG-3 can improve response rates but increase toxicity, highlighting a continued need for approaches that enhance antigen recognition and effective T cell priming without excessive systemic immune activation (5).

IFx-Hu2.0 (pAc/Emm55) is a plasmid DNA product that encodes a fragment of the highly antigenic Emm55 M-like protein from *Streptococcus pyogenes* (7). The selection of Emm55 for IFx-Hu2.0 was based on its strong antigenicity for immunotherapeutic purposes and favorable safety profile (8). The concept is that intratumoral transfection and local Emm55 expression provide an immune-priming signal that promotes recognition of tumor-associated antigens within the same lesion. In murine B16 melanoma, intralesional IFx-Hu2.0 delayed tumor growth, increased intratumoral T cell infiltration, and required both CD4^+^ and CD8^+^ T cells (7). Combining IFx-Hu2.0 with anti–PD-1 further improved tumor control and systemic antitumor immunity (7). In a first-in-human study of unresectable stage III/IV melanoma, intralesional IFx-Hu2.0 was feasible, well-tolerated, and induced peripheral antibody responses consistent with immune priming (8). Patients previously refractory to anti–PD-1-based therapy later demonstrated clinical responses upon retreatment with anti–PD-1–based regimens (8). However, the innate immune sensing of Emm55 and the dendritic cell functions it enhances remain poorly defined. Prior work implicated MyD88-dependent signaling in Emm55-driven T cell activation (7). However, the upstream receptor requirements and their relationship to DC cross-presentation should be addressed to optimize this platform and guide rational combinations with immune checkpoint inhibitors (7, 8).

In this study, we assessed tumor control, immune infiltration, and systemic antibody responses. Mechanistically, we examined dendritic cell antigen uptake and cross-presentation using bone marrow–derived DCs from wild-type, MyD88-, TLR2-, and TLR7-deficient mice and quantified CD8^+^ T cell priming using OT-I functional readouts following exposure to Emm55- or empty plasmid-transfected melanoma lysates. We further evaluated the combination of IFx-Hu2.0 with immune checkpoint blockade by comparing anti-PD-1 monotherapy to combination immune checkpoint blockade (anti-PD-1 + anti-CTLA-4 or anti-PD-1 + anti- LAG-3).

## Methods

### Mice

C57BL/6J wild type (WT), MyD88 knockout (MyD88 KO), TLR2 knockout (TLR2 KO), TLR7 knockout (TLR7 KO), and OT-1 transgenic mice (C57BL/6-Tg (TcraTcrb) 1100Mjb/J) were purchased from the Jackson Laboratory and housed at the Animal Research Facility of the H. Lee Moffitt Cancer Center and Research Institute (Tampa, FL). All experiments were performed in accordance with protocols approved by the Institutional Animal Care and Use Committee (IACUC) and the institution’s guidelines. Female mice aged 6–10 weeks were used for all studies. Mice were observed daily and humanely euthanized by CO2 inhalation (CO2 at a displacement rate of 30-70% of the chamber volume/minute per institutional requirements) at the study endpoint.

### Cell lines and culture conditions

Melanoma cells (B16-D5, YUMM 1.7), EL4 thymoma cells were purchased from ATCC. B16-D5 and EL4 thymoma cells were maintained in complete RPMI 1640 medium, whereas YUMM1.7 were maintained in complete DMEM, as described previously (7, 9). B16-D5, herein denoted B16, is a poorly immunogenic and highly metastatic subclone of B16 melanoma, while YUMM 1.7 (Yale University Mouse Melanoma), herein denoted YUMM, is a mouse melanoma cell line that is syngeneic to C57BL/6 and has well-defined human-relevant driver mutations (9, 10). M05, kindly provided by Dr. Kenneth Rock (Dana-Farber Cancer Institute), is an OVA-expressing B16 melanoma cell and was maintained in selection medium containing 0.8 mg/mL of the antibiotic G418 (7).

For antibody response assays, EL4 cells were transfected with pAc/Empty (herein also denoted Empty) or pAc/Emm55 (herein also denoted IFx-Hu2.0) plasmid using Lipofectamine 2000 Reagent (Invitrogen, Carlsbad, CA). The expression of Emm55 in Emm55-transfected EL4 cells was confirmed by qRT-PCR.

### Melanoma tumor models

Mice were inoculated subcutaneously (s.c.) in the flank with 1 × 10^5^ B16-D5 melanoma cells (day -8). On day -1, palpable tumor-bearing mice received intralesional injections of 20 µg of pAc/Empty vector or pAc/Emm55 vector at weekly intervals for 2 weeks. Invivo-jetPEI transfection reagent (VWR, Radnor, PA) was mixed with pAc/emm55 or pAc/Empty vector (provided by TuHURA Biosciences Inc.) to prepare a homogenous complex for intratumoral injection. A group of tumor-bearing mice that remained untreated served as the control. Treatment with checkpoint blockade therapies was started on day 0. Mice were intraperitoneally injected twice weekly of either isotype IgG control (250 µg), anti-PD-1 (250 µg), anti-CTLA-4 (300 µg), anti-LAG-3 (300 µg), or combination. Tumor growth was measured 2–3 times per week using digital calipers. Tumor volumes were calculated using the formula V = (L x W x W)/2, where V is tumor volume, W is tumor width, and L is tumor length. Tumor growth curves were analyzed using the CGGC permutation test as previously described (11). Animal groups were randomized prior to allocation to treatment groups. Treatment administration and tumor measurements were performed by separate scientists to reduce potential bias in outcome assessment.

### Antibody response assays

Sera were collected from murine peripheral blood on day 7 or day 14 after vector treatment. For antibody responses to pAc/Emm55 plasmid, sera collected from Empty- or Emm55-treated mice were incubated for 24 hours with Emm55-transfected EL4. For antibody responses to melanoma, sera collected from Empty- or Emm55-treated mice were incubated for 24 hours with respective melanoma cell lines (B16 or YUMM cells). The cells were then washed and stained with fluorescent-conjugated anti-mouse IgG antibody (Biolegend, San Diego, CA) and measured on the BD LSR II cytometer (BD Biosciences, San Jose, CA). Fluorescence bound to the cells were measured using flow cytometry. Untreated cells, not incubated with serum, served as controls.

### Bone marrow–derived dendritic cells

Bone marrow cells were flushed from femurs and tibias of WT, MyD88 KO, TLR2 KO, or TLR7 KO mice. Red blood cells were lysed with ACK lysis buffer. The cells were then cultured in RPMI 1640 containing 10% FBS, 20 ng/mL recombinant mouse GM-CSF (PeproTech, Rocky Hill, NJ), and 10 ng/mL IL-4 (PeproTech, Rocky Hill, NJ) for 6 days. BMDCs were enriched by Opti-Prep gradient (Alere Technologies AS, Oslo, Norway). For flow cytometry, cells were stained with fluorochrome conjugated antibodies and analyzed after gating on FSC/SSC morphology, singlets (FSC-A/FSCH), and Zombie NIR –negative viable cells (Biolegend, San Diego, CA) on the LSR II cytometry.

### Antigen uptake and antigen-cross-presentation

For antigen uptake, BMDCs were incubated with 10 µg/mL OVA-FITC (Invitrogen, Carlsbad, CA, Cat.# O23020) at 37^0^C for 2 hours. As a control, another set of BMDCs were incubated at 4 °C to inhibit endocytosis. The cells were washed and OVA-FITC uptake was measured.

For antigen cross-presentation, BMDCs were pulsed with 1 mg/mL OVA (Anaspec, Fremont, CA) at 37^0^C overnight to allow antigen processing and presentation. Next day, DC were stained with PE anti-mouse H-2Kb/SIINFEKL (BD, San Jose, CA, Cat.# 570013, Clone 25-D1.16.rMAb) for flow cytometry analysis. Untreated BMDCs were used as a control. All samples were acquired using an LSRII flow cytometry. The data were analyzed using FCS Express software (*De Novo* Software, Pasadena, CA).

### T cell co-cultures and measurement of IFN-γ

M05 tumor cells were transfected with pAc/empty or pAc/emm55 *in vitro*. Cel lysate was generated by 3 cycles of rapid freeze–thawing. Cell lysates were pulsed onto DC at a 3:1 ratio and cultured for 18–24 h. Untouched CD8 T cells were isolated from the spleens of OT-I mice using CD8a+ T Cell Isolation Kit (Miltenyi Biotec, Cat.# 130-104-075). DCs were co-cultured with OT-I T cells at a 1:10 ratio for 24 h, and supernatants were collected. IFN-γ was measured in supernatants using an IFN-γ ELISA assay kit (BD Biosciences, San Jose, CA) according to manufacturer’s instructions.

### Immunohistochemistry

Immunohistochemistry was performed as a blinded study by the Tissue Core Facility at Moffitt Cancer Center. Tumors harvested on day 9 post-treatment were formalin fixed and paraffin embedded. Sections were stained for anti-mouse CD8 (Cell Signaling, Danvers, MA, Cat.# 98941, Clone D4W2Z, 1:100 dilution) and CD11c (Cell Signaling, Danvers, MA, Cat.# 97585, Clone D1V9Y, 1:200 dilution). Mouse spleen was used as a negative control for immunohistochemistry. Positive cells were quantified using QuPath image analysis software.

### Statistical analysis

Statistical analysis of tumor growth curves was performed using a CGGC permutation test (11). Multiplicity-adjusted p-values were calculated using the Holm method to control the family-wise error rate at 0.05. GraphPad Prism 10 (GraphPad Software, San Diego, California USA) was used for statistical analysis. A one-way ANOVA followed by Tukey’s *post-hoc* test was used to compare serum treatments. A two-way ANOVA followed by Dunnett’s multiple comparisons test was used to assess difference in IFN-γ production between treatments among strains. Statistical significance was achieved at two-sided *p* < 0.05.

## Results

### IFx-Hu2.0 administration suppresses melanoma tumor growth and increases intratumoral immune infiltration

**Figure 1:**
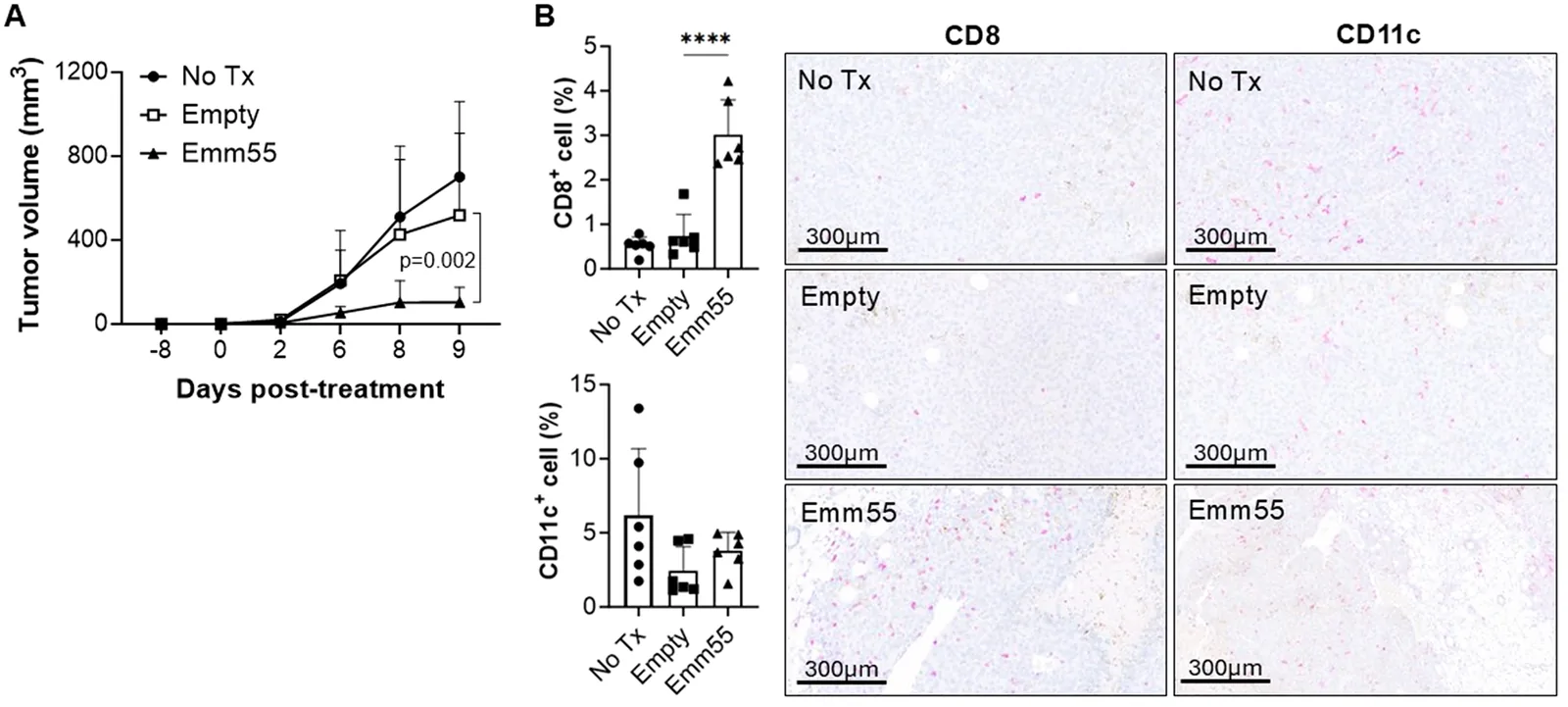
Administration of IFx-Hu2.0 decreases melanoma growth. C57BL/6J mice were sc. injected with 1x10^5^ B16 melanoma cells to induce tumors. Seven days later (day -1), tumors were administrated intralesionally with 20 µg of either nothing (No Tx), Empty plasmid, or Emm55 plasmid. **(A)** Tumor growth curves were analyzed by CGGC permutation test (n=13 mice per group). **(B)** Tumors were collected on day 9 and stained for CD8 and CD11c by immunohistochemistry. Positive cells were quantified using QuPath (left panel). Representative images at 20X magnification are shown in the right panel. ****p<0.0001.

To assess Emm55 effects on melanoma progression, B16 tumors in C57BL/6J mice were treated intralesionally with Empty or Emm55 encoding plasmid (IFx-Hu2.0). IFx-Hu2.0 treatment significantly reduced tumor growth compared with the Empty and no-treatment groups (**Figure 1A**). Consistent with the reduced tumor burden, Emm55-treated tumors exhibited a marked increase in CD8^+^ T cell infiltration, whereas CD11c^+^ dendritic cells remained unchanged (**Figure 1B**).

### IFx-Hu2.0 enhances systemic antibody responses against melanoma and plasmid treatment

To assess humoral immune responses elicited by IFx-Hu2.0 treatment, we evaluated two independent antibody responses: (1) antibodies targeting Emm55 and (2) antibodies recognizing melanoma cells.

**Figure 2:**
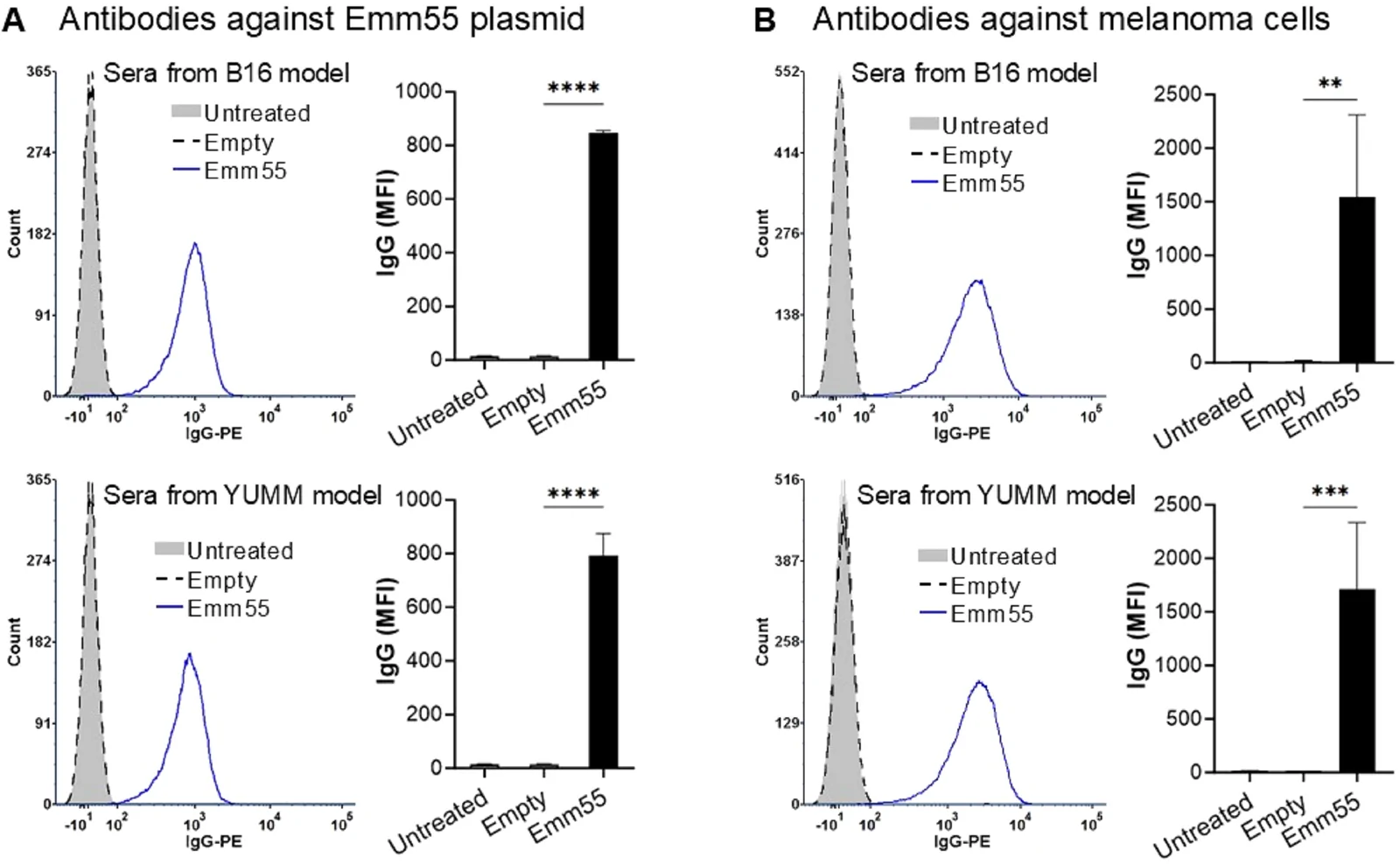
IFx-Hu2.0 treatment enhanced antibody responses to melanoma. B16- or YUMM-bearing mice were treated with Empty or Emm55 plasmid as in **Figure 1**. Serum was collected on day 14 post-treatment. **(A)** Antibodies against Emm55 plasmid. Serum from B16 model (top panels) and YUMM model (bottom panels) were incubated with Emm55-transfected EL4 cells for 24 hours. The same EL4 cells were left untreated as a control. Representative histograms (left panels) and mean fluorescence intensity (MFI, right panels) of IgG bound to the cells were measured using flow cytometry. Serum was from 5 mice per group. An ordinary one-way ANOVA with Tukey’s multiple comparisons test was used to analyze the data, ****p < 0.0001. **(B)** Antibodies against melanoma. Serum from B16 model (top panels) and YUMM model (bottom panels) were incubated with its respective cell lines for 24 hours. A group of respective cell lines was left untreated as a control. Representative histograms (left panels) and mean fluorescence intensity (MFI, right panels) of IgG bound to the cells were measured using flow cytometry. Serum was from 5 mice per group. An ordinary one-way ANOVA with Tukey’s multiple comparisons test was used to analyze the data, **p < 0.01, ***p < 0.001.

To determine whether mice generated antibodies against the Emm55 plasmid, EL4 cells, a non-melanoma cell line, were transfected with an Emm55-encoding plasmid and then incubated for 24 hours with serum from tumor-bearing mice treated with either Empty or Emm55-encoding plasmid. The untreated Emm55-transfected EL4 served as a control. Afterward, a fluorescently tagged secondary goat anti-mouse IgG was used to detect serum antibodies bound to the cells using flow cytometry. Mice treated with Emm55 rapidly produced systemic antibodies against Emm55-expressing cells in both B16 and YUMM melanoma models as early as day 7 post-treatment (**Supplementary Figure 1A**), with significant increases observed by day 14 (**Figure 2A**).

To assess melanoma-specific antibody responses, serum from the same mice was incubated with the corresponding melanoma cells. Antibody binding to B16 or YUMM cells was similarly detected by flow cytometry. Melanoma-specific antibodies were not significantly increased at the early time point (day 7 post-treatment) (**Supplementary Figure 1B**) but were significantly increased by day 14 in serum from Emm55-treated mice (**Figure 2B**). Together, these data indicate that IFx-Hu2.0 induces both plasmid-specific and melanoma-specific humoral responses.

### TLR2 is required for Emm55-mediated enhancement of dendritic cell antigen presentation and T-cell activation

**Figure 3:**
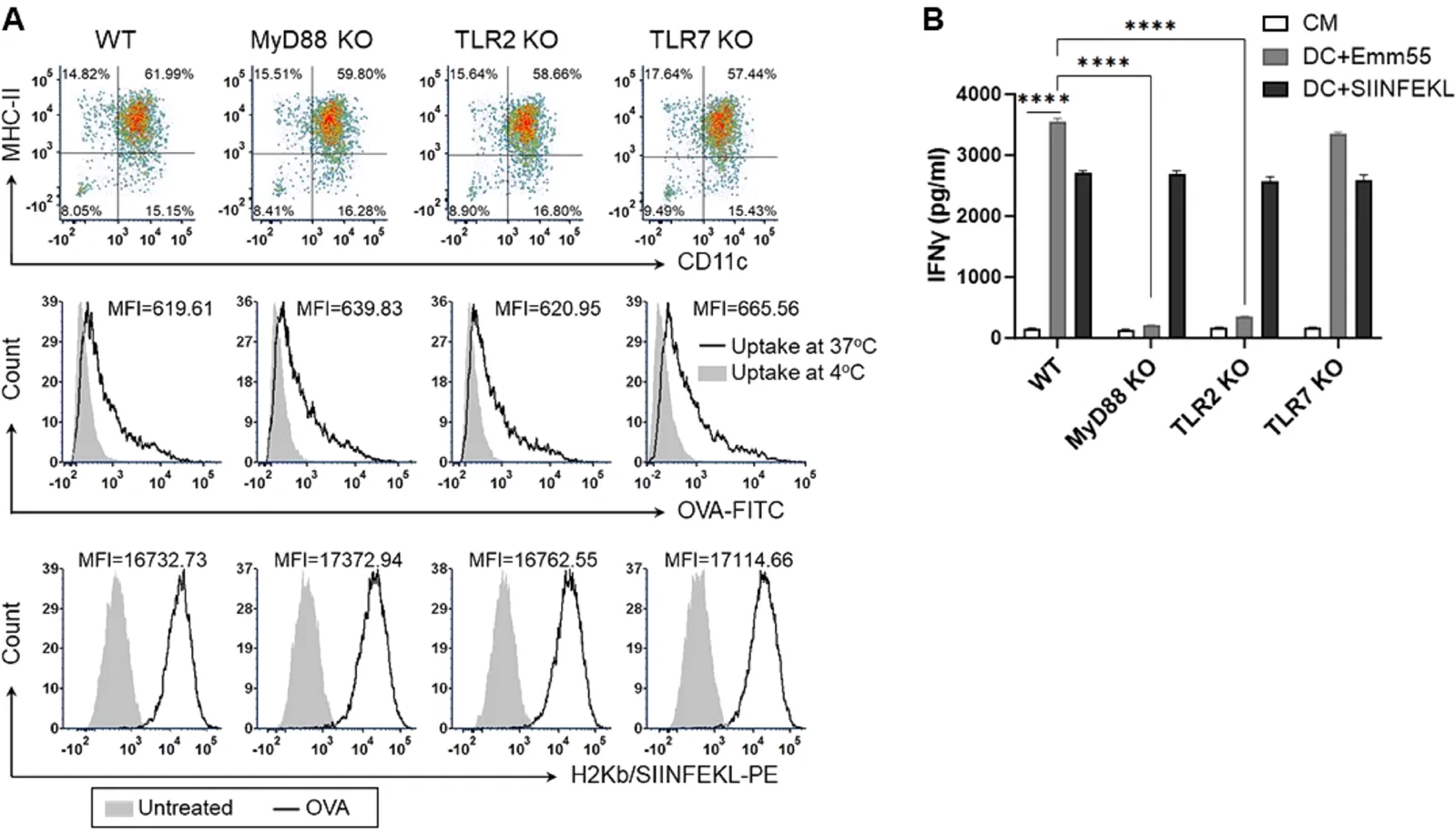
TLR2 is responsible for Emm55-mediated enhanced IFN-γ responses. **(A)** BMDCs from WT, MyD88 KO, TLR2 KO and TLR7 KO mice were characterized using flow cytometry. The cells were first gated based on forward scatter (FSC) and side scatter (SSC), then gated for single live cells by size (FSC-A) and Zombie NIR exclusion. Top panels: to identify DCs, the single viable cells were gated on co-expression of CD11c and MHCII. Middle panels: to access antigen uptake, BMDC were incubated with 10ug/ml OVA-FITC at 37 °C (black line) or 4 °C (filled) for 2 hours. Bottom panels, to access antigen cross-presentation, BMDC were pulsed with 1 mg/mL OVA (black line) or left untreated (filled). Data represents two independent experiments. **(B)** BMDC were pulsed with lysates derived from either Empty- or Emm55-transfected M05 melanoma cells and then incubated with OT-1 T cells (ratio 1:10) for 24 hours. IFN-γ produced by activated T cells in the supernatant was measured. CM = complete medium. N = 6, data from two independent experiments with triplicates for each experiment. A two-way ANOVA with Dunnett’s multiple comparisons test was used to analyze the data, ****p < 0.001.

TLR activation enhances dendritic cell antigen presentation to CD8^+^ T cells and cytokine production (12–14). Our previous work demonstrated that Emm55-induced IFN-γ production by CD8^+^ T cells required MyD88, which is a key protein downstream of most Toll-like receptors (7). To further investigate the mechanism underlying Emm55-mediated immune activation, BMDCs were generated from WT mice and TLRs- or MyD88-deficient mice. The cells were characterized by flow cytometry using CD11c and MHC class II (MHC-II). Cell phenotypes of CD11c^+^MHC-II^+^ BMDCs across WT, MyD88-KO, TLR2-KO, and TLR7-KO genotypes were similar (**Figure 3A**, top). OVA antigen uptake (**Figure 3A**, middle) and OVA antigen cross-presentation (**Figure 3A**, bottom) revealed that deficiency of TLR2, TLR7, or MyD88 signaling on BMDCs did not affect their ability to uptake and present antigens.

To assess Emm55 effects on dendritic cell cross-presentation and CD8^+^ T cell activation, M05 cells were transfected with Empty or Emm55 vectors and lysed by three freeze–thaw cycles. BMDCs were pulsed with the cell lysates and then incubated with OT-I T cells. BMDCs pulsed with Emm55-transfected melanoma lysates significantly induced IFN-γ production by OT-I cells (**Figure 3B**). This enhancement was lost in TLR2- and MyD88-deficient BMDCs, but not in TLR7-deficient BMDCs, indicating a requirement for TLR2/MyD88 signaling in Emm55-mediated T-cell activation.

### IFx-Hu2.0 combined with immune checkpoint therapies further suppress melanoma growth

**Figure 4:**
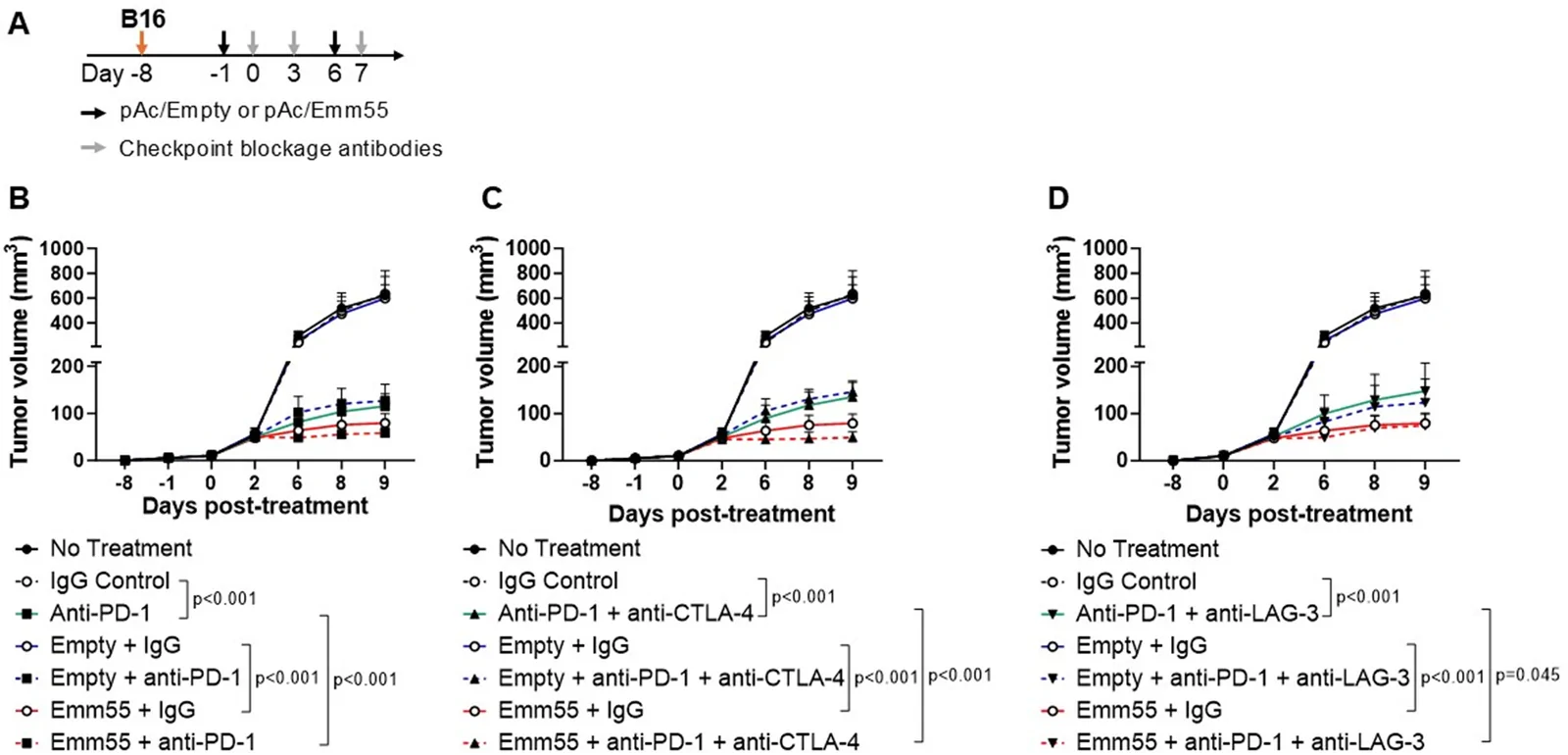
Combination of IFx-Hu2.0 treatment and checkpoint therapies improved anti-tumor activities. **(A)** Treatment diagram in B16 melanoma model. C57BL/6J mice were sc. injected with 1x10^5^ B16 melanoma cells to induce tumors. Empty plasmid or Emm55 plasmid (20 µg/mouse) were injected intralesionally once weekly. Antibodies IgG (250 µg), anti-PD-1 (250 µg), anti-CTLA-4 (300 µg), and anti-LAG-3 (300 µg) were intraperitoneally administered twice weekly. Tumor growth curves of mice treated with Emm55 and anti-PD-1 **(B)** or with Emm55 and checkpoint combination **(C, D)**. Data was analyzed using CGGC permutation test. n = 10 mice per group, from two independent experiments.

Given the robust immune activation elicited by IFx-Hu2.0, we next evaluated whether IFx-Hu2.0 could enhance the activity of checkpoint inhibitors in B16 melanoma model. The treatment diagram is summarized in **Figure 4A**. Either anti-PD-1 therapy or single agent IFx-Hu2.0 treatment markedly reduced tumor growth compared to control (**Figure 4B**). Moreover, the combination of IFx-Hu2.0 and anti-PD-1 treatment was more effective than anti-PD-1 monotherapy (p<0.001) (**Figure 4B**). Dual-checkpoint therapies (anti-PD-1 + anti-CTLA-4 and anti-PD-1 + anti-LAG-3) did not significantly improve tumor inhibition compared with single anti-PD-1 administration; **Supplementary Figure 2**). Consistent inhibition of tumor progression was shown across IFx-Hu2.0 and dual-checkpoint combinations (**Figures 4C, D**).

## Discussion

We investigated how the intratumoral plasmid DNA product IFx-Hu2.0 (pAc/Emm55), encoding a *Streptococcus pyogenes*–derived Emm55 fragment, drives anti-melanoma immunity and enables rational combination with immune checkpoint blockade. Using the B16 melanoma model, we demonstrated that Emm55 treatment suppressed tumor growth, increased intratumoral CD8^+^ T cell infiltration. We previously showed that IFx-Hu2.0 markedly induced interferon signaling pathways (8), a driver of CD8 T cell cytotoxicity including the upregulation of granzyme B expression and IFN-γ production. Interestingly, intratumoral abundance of CD11c^+^ dendritic cells was not altered, suggesting that Emm55 does not simply increase myeloid cell recruitment but rather enhances functional antigen presentation and effector T-cell priming within the tumor. Emm55 enhanced DC-dependent CD8^+^ T cell priming, as illustrated by increased OT-I IFN-γ production following DC exposure to lysates from Emm55-transfected melanoma cells. This effect was TLR2 but not TLR7 dependent, identifying a TLR2–MyD88 axis underlying Emm55 activity in dendritic cells. Our data support a TLR2–MyD88–dependent mechanism in DCs based on ex vivo findings; however *in vivo* validation remains to be established. Conditional DC-specific knockout models are necessary to definitively establish this mechanism *in vivo*. More therapeutic models as well as survival and toxicity analyses need to be explored to validate the immune-priming effect of IFx-Hu2.0 and the role of DCs in this context.

Administration of IFx-Hu2.0 induced systemic IgG binding to Emm55-expressing EL4 and melanoma cells. This binding is specific as supported by multiple control conditions (**Supplementary Figure 3**). Antibody isotype(s) responsible for the binding remain undefined and represent an important area for future investigation.

Finally, we found that combining intratumoral Emm55 with PD-1 blockade provides improved tumor control compared with either treatment alone, whereas adding CTLA-4 or LAG-3 blockade to anti–PD-1 did not confer additional benefit in this setting. Together, these data support Emm55 as an *in situ* immune-priming strategy to combine with PD-1 blockade in melanoma. Dual immune checkpoint blockade is associated with upwards of 50% severe multi-organ immune-related toxicities leading to treatment discontinuation and long-term morbidity (15–17). Intratumoral Emm55 therapy represents an alternative immunotherapeutic strategy due to its minimal toxicity (8). Emm55 is sufficiently immunogenic to prime the immune system while its intratumoral administration confines initial immune activation to the tumor microenvironment. Importantly, we demonstrated that anti-tumor activities of anti-PD-1/Emm55 combination is sufficient to match or exceed the efficacy observed with dual immune checkpoint blockade regimens such as anti-PD-1/anti-CTLA-4 or anti-PD-1/anti-LAG-3. Combining Emm55 with single-agent PD-1 blockade offers an effective, lower-toxicity therapeutic strategy for clinical trials.

Several dendritic cell (DC)–mediated mechanisms have been demonstrated to support cytotoxic CD8^+^ T-cell immunity, including: (1) enhanced cross-presentation of exogenous antigens to potentiate naïve CD8^+^ T-cell priming; (2) upregulation of costimulatory molecules on DCs to enable full CD8^+^ T-cell activation; and (3) DC-derived cytokine production, such as IL-12 and type I interferons, which promotes CD8^+^ T-cell expansion, differentiation, and metabolic programming (18–21). TLR2 signaling has been implicated in regulating several of these DC functions, including antigen cross-presentation and inflammatory cytokine production (13, 14), suggesting enhanced antigen presentation on DCs following Emm55 treatment as a possible mechanism to activate T cells and enhance its cytokine production because this is a well-established pathway mediated through TLR2 in DCs (22). The specific mechanisms by which Emm55 mediates DC-driven cytotoxic CD8^+^ T-cell priming and promotes IFN-γ production were not explored in this brief report and are topics for further investigation. However, this brief report and others suggest that the MyD88-TLR2 axis is important for this effect.

There are over 200 *Streptococcus pyogenes* emm types that have been identified, with certain emm types preferentially associated with invasive versus non-invasive disease phenotypes (23). Emm55 represents one member of this diverse family of M proteins, which are highly variable at the N-terminal region and define serotype specificity (24). IFx-Hu2.0 encodes only a fragment of the Emm55 protein specifically engineered as a cell surface–anchored M protein rather than a secreted exotoxin. It was chosen to trigger the immune system to increase the inflammatory response, leading to antigen presentation (7). The properties of the Emm55 fragment avoid the rheumatological side effects such as Rheumatic fever. Moreover, no dose-limiting toxicities attributable to IFx-Hu2.0 were observed in clinical trials (8). In our preclinical studies, no significant rash was seen in treated animals. Mice treated with intralesional IFx-Hu2.0 improved overall survival compared to control-treated animals (7). Therefore, the selection of Emm55 for IFx-Hu2.0 was based on its strong antigenicity for immunotherapeutic purposes and favorable safety profile.

The therapeutic rationale for IFx-Hu2.0 is supported by preclinical and early clinical evidence demonstrating its immune-priming activity. In murine melanoma models, intralesional IFx-Hu2.0 induces tumor growth delay, increases CD4^+^ and CD8^+^ T-cell infiltration, and improves T cell–dependent survival, with further enhancement in combination with PD-1 blockade (7). In horses, intratumoral administration of the Emm55 plasmid vaccine produces a systemic antitumor response, with ~42% tumor regression - including noninjected lesions, and a sustained increase in antimelanoma IgG antibodies (25). The first-in-human study shows that IFx-Hu2.0 is well tolerated and activates both innate and adaptive immune responses, including interferon pathway activation and IgG/IgM responses against Emm55 and melanoma-associated antigens (8). Notably, three of four patients previously refractory to anti-PD-1 experienced clinical benefit upon subsequent anti-PD-1–based therapy, suggesting conversion of immunologically “cold” tumors to more responsive “hot” tumors (8). Although preliminary and based on small cohorts, these findings provide a coherent biological rationale for further study, particularly in combination with checkpoint blockade, and highlight the need for larger trials to define optimal clinical use.

In conclusion, Emm55 acts as a potent intratumoral immune-priming agent through a TLR2–MyD88–dependent dendritic cell mechanism, supporting its clinical evaluation in combination with PD-1 blockade.

## Data Availability

Data supporting the findings of this study are publicly available in the Immunology Database and Analysis Portal under study accession SDY3724. Please contact the corresponding author for other manuscript requests.
